# Correction: Zhao et al. Effects of Different Feed Additives on Intestinal Metabolite Composition of Weaned Piglets. *Metabolites* 2024, *14*, 138

**DOI:** 10.3390/metabo16080521

**Published:** 2026-07-24

**Authors:** Mingxuan Zhao, Jian Zhang, Fuzhou Liu, Lv Luo, Mingbang Wei, Yourong Ye, Chamba Yangzom, Peng Shang

**Affiliations:** 1College of Animal Science, Tibet Agriculture and Animal Husbandry University, Linzhi 860000, China; m19863998873@163.com (M.Z.); zhj88n@126.com (J.Z.); liufuzhou2022@163.com (F.L.); ll202300201095@163.com (L.L.); bangbangbang962@163.com (M.W.); yeyourong@xza.edu.cn (Y.Y.); 2The Key Laboratory of Tibetan Pigs Genetic Improvement and Reproduction Engineering, Linzhi 860000, China; 3The Provincial and Ministerial Co-Founded Collaborative Innovation Center for R & D in Tibet Characteristic Agricultural and Animal Husbandry Resources, Linzhi 860000, China

## References

The authors would like to make the following correction to their published paper [1].

The change is as follows:

Reference [44] in the original publication is incorrectly cited and needs to be replaced with the following reference:44.Yang, D.; Zhang, S.; Fang, X.; Guo, L.; Hu, N.; Guo, Z.; Li, X.; Yang, S.; He, J.C.; Kuang, C.; et al. *N*-benzyl/aryl substituted tryptanthrin as dual inhibitors of indoleamine 2,3-dioxygenase and tryptophan 2,3-dioxygenase. *J. Med. Chem.* **2019**, *62*, 9161–9174.

The authors state that the scientific conclusions are unaffected. This correction was approved by the Academic Editor. The original publication has also been updated.
